# A Two-House Field Comparison of Multi-Parameter and Conventional Environmental Control for Rabbits Under Hot–Humid Summer Conditions

**DOI:** 10.3390/ani16213450

**Published:** 2026-11-01

**Authors:** Quan-Gang Wang, Qian Jing, Guoxin Wu, Hang Shu, Zhong-Ying Liu, Ronghua Ji, Yao Guo, Zhong-Hong Wu

**Affiliations:** 1 State Key Laboratory of Animal Nutrition and Feeding, College of Animal Science and Technology, China Agricultural University, Beijing 100193, China; pftzdwyzc@163.com (Q.-G.W.); s20243040881@cau.edu.cn (Q.J.); shuhang@cau.edu.cn (H.S.); lzy228@cau.edu.cn (Z.-Y.L.); 2 College of Information and Electrical Engineering, China Agricultural University, Beijing 100083, China; wugx22@163.com (G.W.); jessic1212@cau.edu.cn (R.J.)

**Keywords:** rabbit housing, evaporative cooling, multi-parameter control strategy, hot–humid climate, physiological and growth performance, cooling pad recirculation, electricity use, economic analysis

## Abstract

This study compared a programmable logic controller (PLC) combining indoor temperature, outdoor temperature, and outdoor relative humidity with conventional temperature-triggered pad cooling and manual fan adjustment in two commercial rabbit houses in Leshan, China, a region characterized by a hot and humid climate. The 33-day summer trial included 108 selected Hyla rabbits aged 39 d per house. The PLC house had a mean air temperature 0.36 °C higher and relative humidity 4.34 percentage points lower than the control house. Final body weight and average daily gain were higher, while feed conversion did not differ significantly. Intra-abdominal temperature near the cooling pads was 0.29 °C lower. Estimated cooling pad recirculation was 81.0% lower and combined fan and pump electricity use was 21.2% higher. A partial-budget analysis at a standardized scale of 3000 retained rabbit production-equivalents estimated a benefit 2955.95 Chinese yuan (CNY) higher for the treatment house than for the control house under the specified prices and scaling assumptions. Each strategy was applied in one house, so these preliminary differences cannot be separated from house effects. These observations support further evaluation in independently replicated houses, with animal responses, resource use, and deployment costs assessed together.

## Simple Summary

Hot and humid weather presents a challenge for cooling commercial rabbit houses. We compared a controller using indoor temperature and outdoor temperature and humidity with conventional temperature-triggered pad operation and manual fan adjustment over 33 summer days. Each house included 108 selected rabbits for study measurement. The multi-parameter house had lower relative humidity, greater feed intake and growth, and lower intra-abdominal temperature near the cooling pads. Feed conversion did not differ significantly. Cooling pad water recirculation was lower, whereas combined fan and pump electricity use was higher. The meters measured circulated water rather than fresh water consumption. As each strategy was used in one house, the findings provide preliminary field evidence. These findings highlight the value of considering animal responses, resource use, and deployment costs together when selecting a cooling strategy.

## 1. Introduction

Projected warming is expected to increase heat stress exposure across livestock production systems, threatening animal welfare, productivity, and resource efficiency [1,2,3]. Growing rabbits warrant particular attention because their functional sweating capacity is limited and they rely on respiratory and auricular heat dissipation [2]. When high air temperature coincides with high relative humidity, the reduced vapor pressure gradient restricts evaporative heat loss. Prolonged heat exposure can therefore elevate body temperature, reduce voluntary feed intake and growth, disrupt metabolic and immune homeostasis, and increase production losses [2,4,5,6,7,8]. Environmental control in commercial rabbit houses must therefore account for interacting climatic factors rather than treating indoor temperature as the only relevant signal.

Negative-pressure ventilation combined with fan pad evaporative cooling is widely used to remove sensible heat and reduce the dry bulb temperature of incoming air. Control based solely on indoor temperature initiates the corresponding cooling response once a set point is reached. During rapid weather changes, thermal inertia and delays in air circulation and equipment response may cause control to lag behind changing conditions [9]. Incorporating outdoor temperature and relative humidity allows evaporative cooling potential to be assessed from current conditions alongside indoor cooling demand [10]. Control decisions can therefore account for changing outdoor conditions as well as indoor feedback, providing a basis for more timely equipment adjustments and a more stable indoor environment [10,11]. This approach warrants particular attention in hot and humid climates, where evaporative cooling is constrained and pad operation adds moisture [12]. Weather-based ventilation optimization and coordinated ventilation control offer potential to improve temperature regulation and energy efficiency in livestock and poultry housing [11,13]. However, whether these improvements justify additional outdoor sensors, maintenance, and control complexity depends on their benefits under production conditions.

Experimental evidence shows that heat stress impairs rabbit growth, whereas improved ventilation can reduce body temperature and increase milk yield in dairy cows [6,14]. Nevertheless, large-scale dairy production data indicate that cooling infrastructure and management adjustments only partially offset losses associated with humid heat [15]. Cooling design also affects resource use: simulations of supplemental cooling in dairy buildings indicate potential energy savings, while dairy farms using less spray water can consume more electricity per unit of milk [16,17]. Environmental improvements, animal benefits, and resource savings therefore require joint evaluation within the same production setting. Previous studies have verified the cooling effect of multi-parameter control in rabbit houses against manually controlled houses in high-temperature and low-humidity regions [10]. However, the performance of this multi-parameter strategy combined with intermittent cooling pad watering remains to be verified under hot and humid conditions.

We developed a programmable logic controller (PLC) with multi-parameter control logic and conducted a concurrent two-house field comparison against conventional temperature-triggered pad cooling with manual fan adjustment during a hot–humid summer in Leshan, Sichuan Province, China. The study contributes the following:

- A field comparison of multi-parameter PLC control combining indoor temperature, outdoor temperature, and outdoor relative humidity with conventional temperature-triggered pad cooling under hot–humid summer conditions.
- An independently developed control system integrating environmental sensing, equipment control, feedback, and alarms.
- A joint assessment of environmental conditions, rabbit temperature and growth, resource use, and partial-budget outcomes.

## 2. Materials and Methods

### 2.1. Control System Design

#### 2.1.1. Hardware and Signal Interfaces

**Figure 1:**
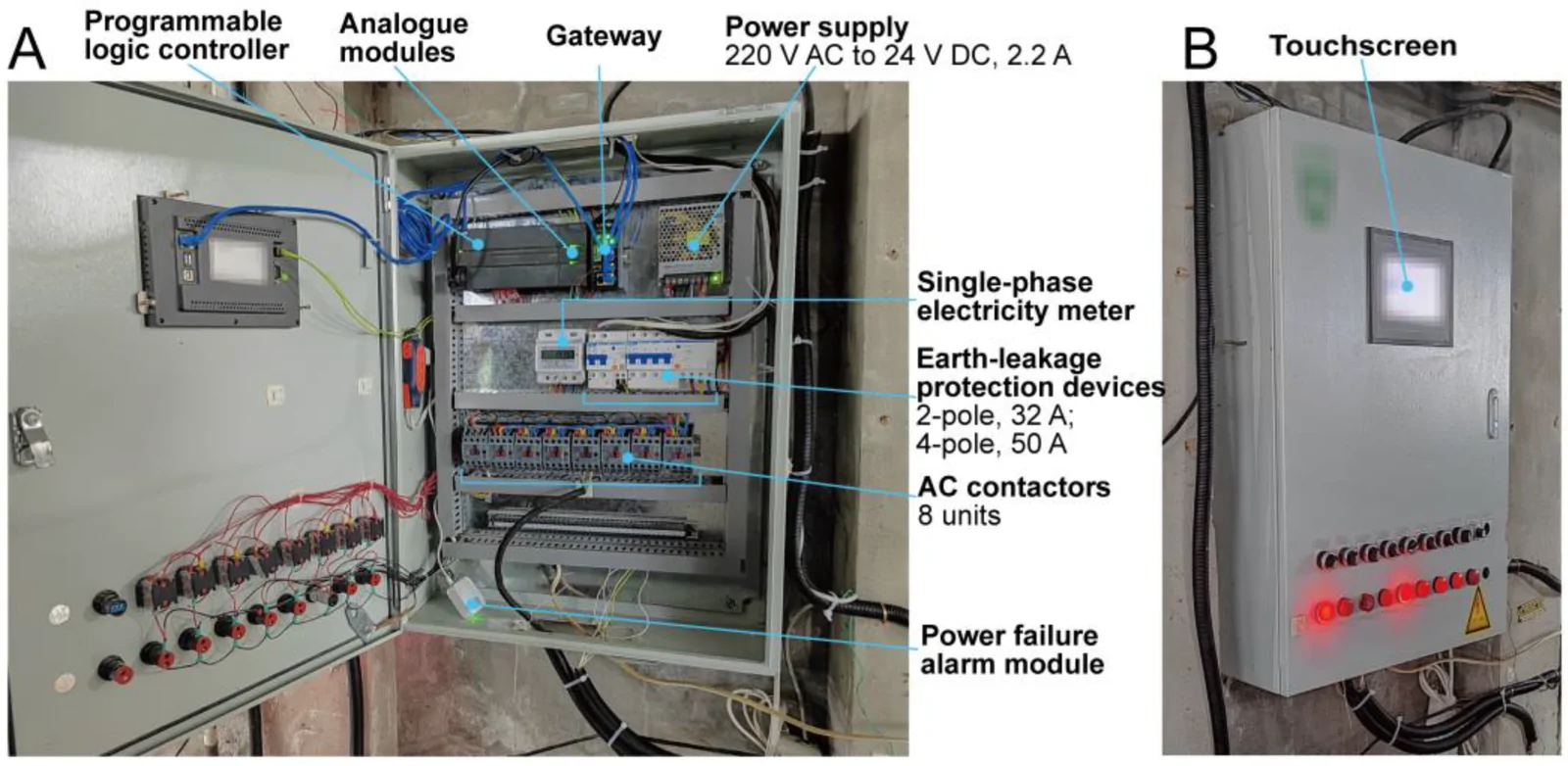
Internal layout and exterior view of the rabbit house environmental control cabinet. (**A**) Internal components; (**B**) Exterior view of the installed cabinet. AC, alternating current; DC, direct current. Non-English content has been obscured using pixelation.

A PLC-based environmental control system was developed for rabbit houses. The hardware consisted of the PLC, environmental sensors, alternating current (AC) contactors, a touchscreen, a communication gateway, and power supply and protection components (Figure 1). A Siemens S7-200 SMART SR60 PLC (Siemens AG, Nuremberg, Germany) interfaced with analogue signals through expansion modules. In the treatment house, sensor measurements were transmitted to the PLC using the Modbus remote terminal unit (RTU) protocol over a Recommended Standard 485 (RS-485) serial network. The RS-485 network supported up to 32 sensor devices. In this study, three indoor sensors and one outdoor sensor measured temperature and relative humidity (RH), while carbon dioxide (CO_2_) concentration was monitored at three indoor locations (Table 1).

PLC digital outputs drove eight AC contactors to switch pumps, heating equipment, and other on/off devices. A variable-frequency drive (VFD) received analogue commands from PLC expansion modules and adjusted its output frequency to regulate fan speed. Equipment status signals returned through PLC digital or analogue inputs for supervision and alarms. The system provided two operating modes, automatic and manual. In automatic mode, the cooling pad pump and variable-speed fans were controlled according to the programmed PLC logic, whereas manual mode allowed operators to control the equipment directly on site.

A 7-inch MCGS TPC7062Ti touchscreen (Shenzhen Kunlun Tongtai Technology Co., Ltd., Shenzhen, China) served as the local terminal and communicated with the PLC via Ethernet. A fourth-generation (4G) cellular/Ethernet gateway linked the local control system to the cloud platform. Electrical protection was provided by 2-pole and 4-pole earth leakage protection devices rated at 32 and 50 A, respectively. A power supply converted 220 V AC to 24 V direct current (DC), with a rated output current of 2.2 A, to power low-voltage devices.

#### 2.1.2. Control and Communication Architecture, Actuator State Feedback, and Alarm Design

**Figure 2:**
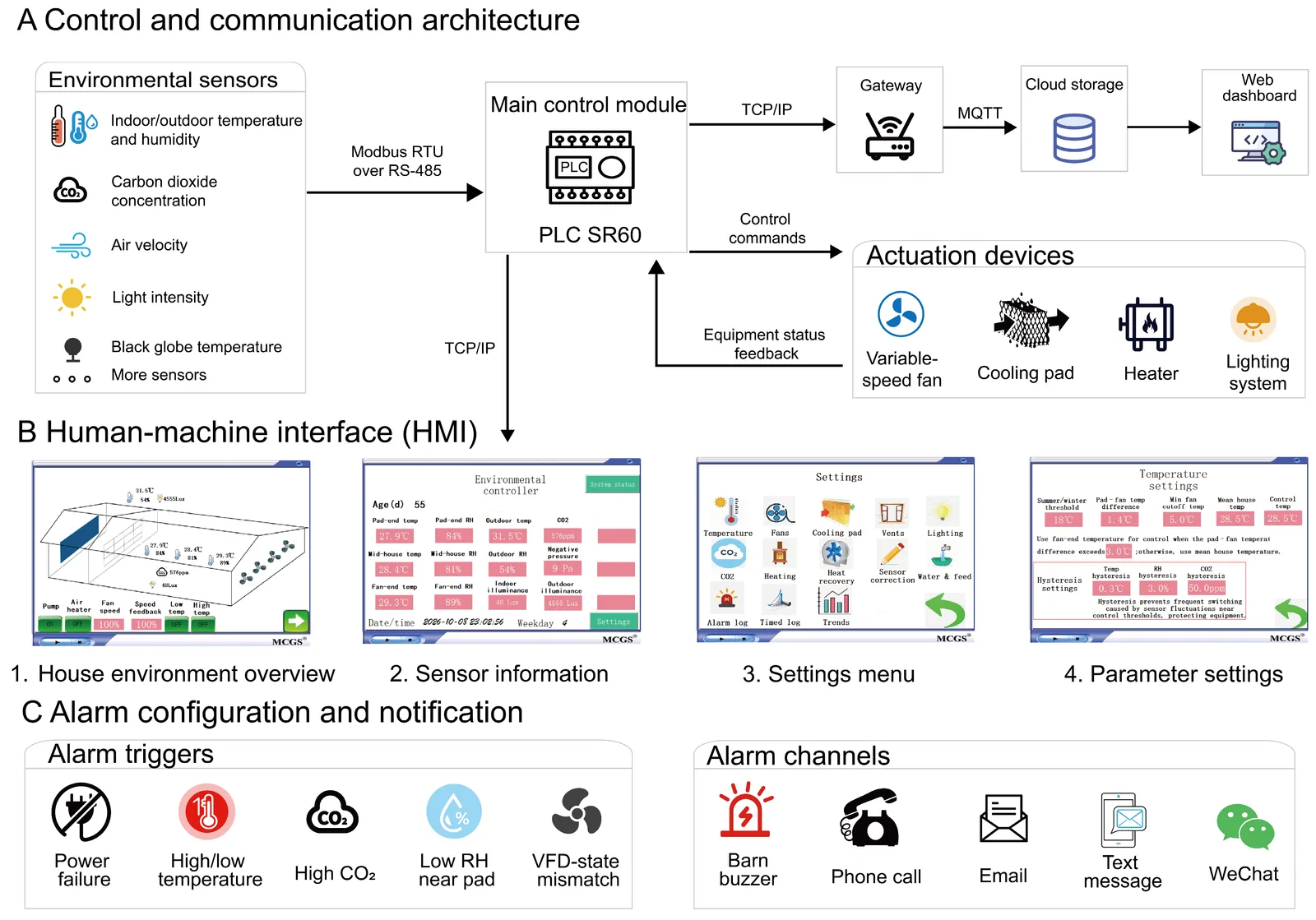
Architecture and functions of the rabbit house environmental control system. (**A**) Control and communication architecture. (**B**) Human–machine interface; the displayed screens are examples generated using the screen simulator. (**C**) Platform alarm categories and notification channels, all functionally tested before the trial. Documented configuration rules are described in Section 2.1.2. PLC, programmable logic controller; HMI, human–machine interface; RH, relative humidity; VFD, variable-frequency drive; CO_2_, carbon dioxide; RS-485, Recommended Standard 485; RTU, remote terminal unit; TCP/IP, Transmission Control Protocol/Internet Protocol; MQTT, Message Queuing Telemetry Transport.

The PLC program was developed using Siemens STEP 7-Micro/WIN SMART V2.7. A main program called subroutines for data acquisition, equipment control, feedback processing, and alarm evaluation. The PLC processed indoor and outdoor environmental data, issued control commands, and received equipment signals to check operating status and detect abnormalities. A human–machine interface (HMI) supported on-site monitoring and parameter adjustment, while a communication gateway enabled data upload and remote access (Figure 2).

An auxiliary contact returned the cooling pad pump contactor state, and the VFD provided analogue feedback and a fault contact. These electrical signals supported equipment supervision but did not measure delivered airflow or water flow. Alarms for high or low indoor temperature, high CO_2_ concentration, and low RH near the cooling pads provided additional environmental warnings. The low-temperature alarm was intended for winter operation and used a 5 °C threshold, with 3 s trigger and recovery delays. The documented threshold rules used fan-end temperature >32.5 °C and indoor CO_2_ >2000 ppm, each with 3 s trigger and recovery delays. The cooling pad low-RH warning used a 70% RH threshold and the same 3 s trigger and recovery delays. The VFD rule, labeled low-speed feedback, uses a frequency feedback threshold <15 Hz with 3 s trigger and recovery delays.

For these documented threshold rules, activation required the condition to persist continuously for 3 s. Recovery required the condition to remain absent for 3 s; recurrence before recovery continued the same event. Before the trial, all alarm types illustrated in Figure 2C were functionally tested, and notifications successfully reached farm personnel through the local buzzer and remote interfaces. Corresponding trial alarm logs were not retained, so end-to-end response delays and quantitative notification reliability were not evaluated.

#### 2.1.3. Human–Machine Interface and Data Management

The HMI was developed using MCGS Embedded V7.7 and included house overview, sensor information, settings, and parameter pages (Figure 2B). Operators could view environmental measurements and equipment status, adjust control parameters, and access historical trends and alarm records.

The touchscreen could store approximately 7 d of data, while the gateway’s memory card provided 32 gigabytes (GB) of additional local storage. In addition to these local backups, the gateway uploaded the data to a cloud database for online backup. The cloud platform supported remote access via a web dashboard or a mobile mini-program.

### 2.2. Multi-Parameter and Single-Parameter Fan Pad Control Logic

**Figure 3:**
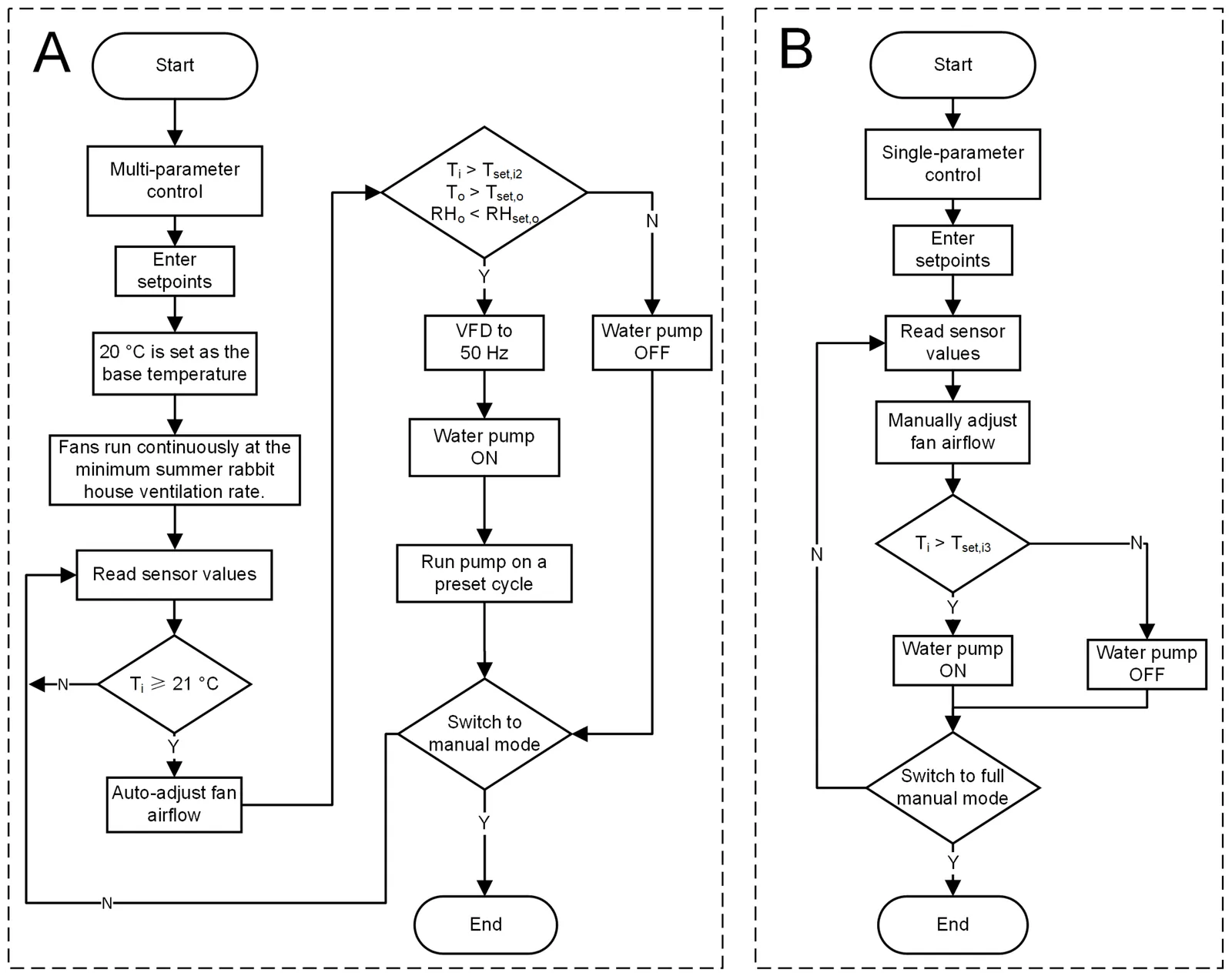
Control logic for (**A**) multi-parameter control and (**B**) temperature-triggered pad cooling with manual fan adjustment. *T_i_* and *T_o_* denote indoor and outdoor temperature, and *RH_o_* denotes outdoor relative humidity. *T_set,i2_* = 26–27 °C, *T_set,i3_* = 26 °C, *T_set,o_* = 27 °C, and *RH_set,o_* = 75% or 82%. The configured maximum fan frequency of 50 Hz precedes pump activation. Pad watering was intermittent (10 min on, 2–4 min off). VFD, variable-frequency drive. Y and N indicate yes and no, respectively.

The treatment strategy used indoor temperature (*T_i_*), outdoor temperature (*T_o_*), and outdoor relative humidity (*RH_o_*) to permit cooling pad operation (Figure 3). Fans were increased first to promote convective cooling before pad operation added moisture. The minimum summer fan command was 30% at *T_i_* ≤ 20 °C and increased by 10 percentage points per 1 °C rise above 20 °C, reaching the configured 50 Hz limit before pump activation. The VFD reached 50 Hz at a controller command of approximately 90%. The outdoor temperature threshold was 27 °C. The outdoor RH threshold increased from 75% to 82% on 9 August, and the indoor temperature threshold decreased from 27 to 26 °C on 15 August to accommodate changing weather and rabbit growth. Pump-on duration remained 10 min. Pump-off duration was 4 min before 11 August and 2 min thereafter. Temperature and RH hysteresis were 0.3 °C and 3 percentage points, respectively.

In the control house, the commercial controller activated the cooling pad pump at an indoor temperature threshold of 26 °C, and farm staff adjusted the fans manually. Cooling pad activation criteria and watering schedules differed between houses. The comparison therefore evaluated their combined operation and did not isolate the contribution of either component.

### 2.3. Experimental Design and Data Collection

The trial ran from 26 July to 28 August 2024 at a commercial rabbit farm in Leshan, Sichuan Province, China. The adjacent mechanically ventilated houses had the same dimensions (50 m × 11 m), orientation, and layout, with cooling pads at the inlet and exhaust fans at the opposite end. Both houses used identical axial fans, pumps, and cooling pads. The axial fans measured 1380 × 1380 × 450 mm and used three-phase asynchronous motors (YJD90S-4, Wendeng Jinda Electrical Machinery Co., Ltd., Weihai, China) rated at 380 V, 50 Hz, 1.1 kW, and 2.8 A, with a speed of 1400 r min^−1^. The five-fan configuration had a field-measured total airflow of 121,270.67 m^3^ h^−1^ at 50 Hz, determined from cross-sectional air velocity measurements. One single-phase pump (QDX1.5-32-0.75, Zhejiang Daniu Pump Industry Co., Ltd., Taizhou, China) was rated at 220 V and 0.75 kW, with a nominal flow of 1.5 m^3^ h^−1^. The cooling pads measured 8.4 m × 2.1 m × 0.15 m (length × height × thickness). The pads were visually inspected before the trial. Strategies were assigned to fixed houses: one used the developed PLC system; the other used the farm’s temperature-triggered BMX1804 controller (Henan Baomingxiang Automation Equipment Co., Ltd., Zhengzhou, China) with manual fan adjustment. The study sample comprised 108 rabbits per house, not the full house population.

Loading estimates used 1166 breeding does, 1100 replacement rabbits, 2750 kits and 108 study rabbits in the treatment house, and 4724 rabbits including 108 study animals in the control house. Category-specific reference weights of 4.5, 3.5, 0.06, and 1.8 kg, respectively, and 1.8 kg for control rabbits gave estimated biomasses of 9456.4 and 8503.2 kg. Dividing by the estimated semicircular roof house volume of 3474.6 m^3^ yielded approximate stocking densities of 2.7 and 2.4 kg m^−3^, respectively.

**Figure 4:**
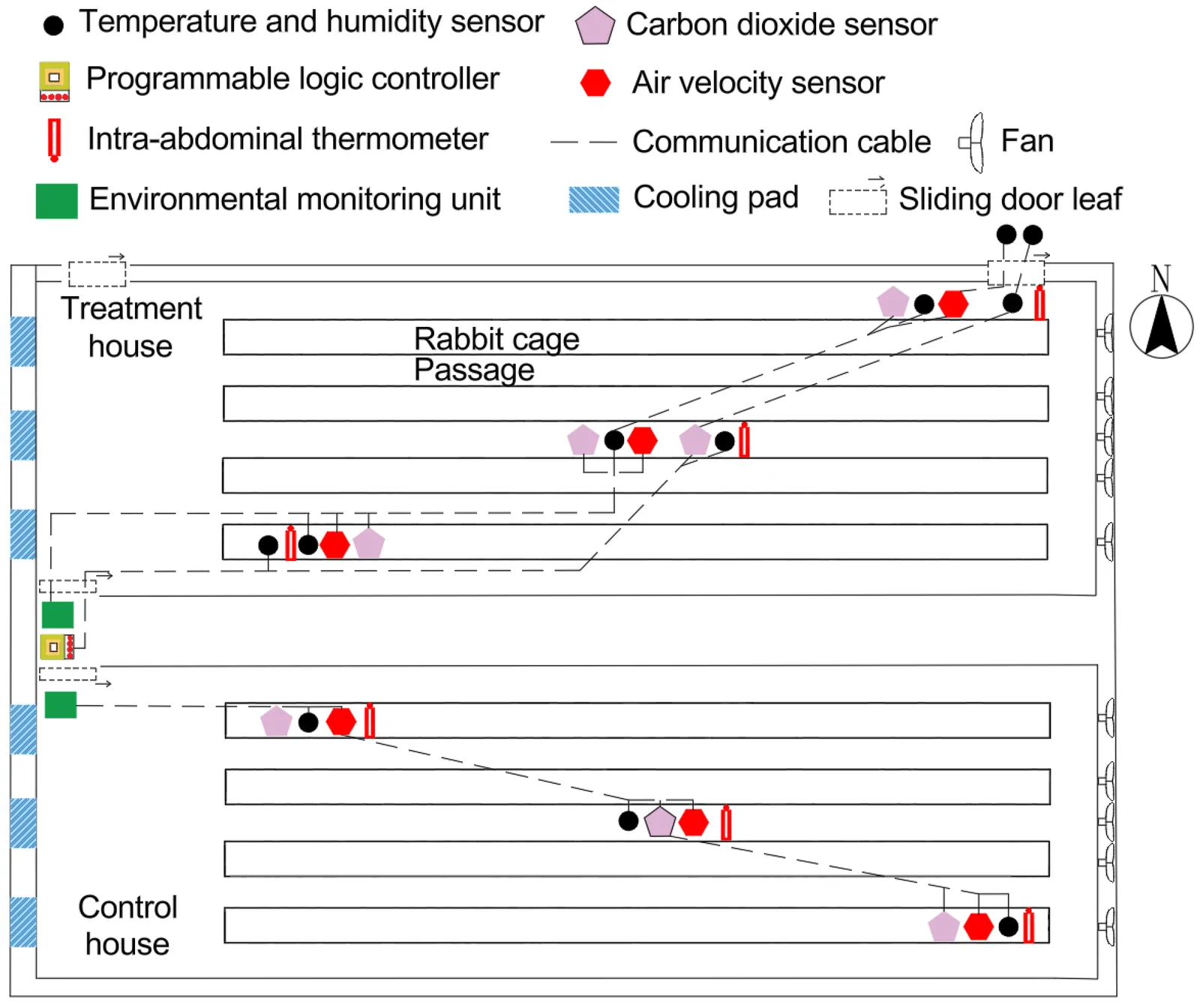
Cooling equipment and environmental and intra-abdominal temperature monitoring locations in the two houses. The cooling pad, central, and fan zones are fixed longitudinal regions. Each thermometer symbol represents a group initially comprising three rabbits selected for implantation. The control house fan zone body temperature series was not included in the primary analysis.

The same environmental monitoring models were installed in both houses at the cooling pad, central, and fan zones, approximately 15 cm above the cages (Figure 4). In each 50 m long house, the cooling pad and central sensors were 3 m and 25 m from the cooling pad, respectively, and the fan zone sensor was 3 m from the fans. The treatment house also had the PLC control sensors. All sensors were new and reported as factory-tested before shipment. Implanted sensors were additionally compared with a mercury thermometer in a water bath. Temperature/RH records were usually collected at 1 min intervals in the treatment house and 5 min intervals in the control house. Implanted temperature records were usually collected every 15 min. Device clocks were synchronized to Beijing time at the start of the trial, and records were aligned by timestamp before analysis. Instrument specifications are listed in Table 1. House-level and zone-level environmental summaries are presented in Table 2 and Table 3, respectively.

On 26 July, 108 mixed-sex Hyla rabbits aged 39 d and of similar body weight were randomly selected from the commercial population in each house and randomly assigned to cage positions across three longitudinal zones, with two rabbits per cage. Retained sample initial weights showed no significant corresponding zone between-house or within-house zone contrasts under the stated procedures (Table 4). Cages measured 54 × 40 × 38 cm (length × width × height). Male and female market rabbits were managed and fed together under routine farm practice; sex was not recorded for the selected animals. Rabbits were weighed on 26 July and 28 August, covering 33 d from 39 to 72 d of age. Nine rabbits per house, initially three per zone, were selected for body temperature sensor implantation.

Both houses used nipple drinkers and the same commercial feed supplied by Rizhao Hengbang Animal Husbandry Technology Co., Ltd., Rizhao, China. Ingredients included maize, soybean meal, bran, oil, alfalfa meal, peanut vine meal, dicalcium phosphate, sodium chloride, choline chloride, vitamin and trace mineral premixes, plant enzymes, mold inhibitors, enzyme preparations, digestive enzymes, and biological preparations. According to the manufacturer’s label, the composition was moisture ≤14%, crude protein ≥16%, crude fiber 7–20%, crude ash ≤14%, calcium 0.6–1.5%, total phosphorus ≥0.4%, sodium chloride 0.3–0.8%, lysine ≥0.6%, and cystine plus methionine ≥0.5%. Feed was offered and weighed at 09:30 daily, with refusals weighed the following morning.

Feed was withdrawn for 8 h before surgery while water remained available. Zoletil 50 (3 mg/kg) and xylazine (1.5 mg/kg) were injected intramuscularly 15 min before surgery. Abdominal hair was clipped, and the skin was disinfected with povidone–iodine and rinsed with sterile saline. The logger was disinfected with alcohol, rinsed with sterile saline, placed intra-abdominally and the incision sutured. Meloxicam (0.2 mg/kg) was given intraoperatively. Postoperative care included enrofloxacin (5 mg/kg) for three days and daily meloxicam (0.2 mg/kg) for three days. Wound healing, body temperature, feeding, drinking, and activity were monitored daily; sutures were disinfected with povidone–iodine, and gauze dressings were changed daily.

Postoperative recovery required body temperature to return to the expected physiological range, feeding and activity to recover, normal posture and grooming to resume, and the surgical wound to remain clean and dry without inflammation. All implanted rabbits met these clinical criteria within 48 h. Body temperature records from the first two postoperative days were excluded, and analysis began two days after implantation. Daily body temperature summaries are presented in Table 5.

Humane intervention criteria included rapid postoperative weight loss, refusal to eat or drink, persistent vocalization, abnormal posture, reluctance to move or social withdrawal, wound infection or dehiscence, sustained fever or low body temperature, or inability to move independently. An affected animal would have procedures suspended, receive veterinary assessment and treatment, and be withdrawn from the experiment. No implanted rabbit died or showed discomfort during the first 48 h after surgery. Implanted rabbits remained excluded from the production analysis.

### 2.4. Water and Electricity Consumption and Economic Benefit Analysis

Water meters were installed horizontally between each sump and cooling pad to measure recirculated flow, and fresh water replenishment was not metered. Water readings spanned 31 July to 28 August 2024, treated as a nominal 28-day interval, and fan and pump electricity readings spanned 28 July to 28 August, a nominal 31-day interval. To use a common 33-day scale, recorded water and electricity totals were multiplied by 33/28 and 33/31, respectively, assuming that the measured mean daily use also applied to unmetered days.

Recorded totals were 388.10 and 2042.40 m^3^ of recirculated water and 4086.04 and 3372.28 kWh of combined fan and pump electricity in the treatment and control houses, respectively. The corresponding daily means were 13.86 and 72.94 m^3^ d^−1^ for recirculation and 131.81 and 108.78 kWh/d for electricity.

Production analysis retained 37 treatment cages (74 rabbits) and 39 control cages (78 rabbits) from 54 two-rabbit cages (108 rabbits) per house. Hierarchical reconciliation assigned excluded cages to implantation (6/6), non-implantation mortality (8/8), diarrhea without implantation or death (1/0), and feed wastage (2/1), respectively (treatment/control). Table S1 provides zone-specific flows and outcome denominators, Figure S1 shows the event timeline, and Table S2 assesses how excluding cages affected by diarrhea or feed wastage influences growth and feed intake estimates. All production records from each excluded cage, including the cage mate, were removed for the full trial. Illness- and mortality-affected cages were excluded because their feeding and growth were not representative of the retained production sample; no feed from these cages entered the reported daily feed intake (DFI) or feed conversion ratio (FCR). Excluded cage counts are not death counts. Eight non-implanted rabbits died in eight treatment cages, while nine died in eight control cages because two rabbits died in one cage. The mean feed intake and weight gain of the retained sample were scaled to a standardized 3000-rabbit scenario for comparison, with a separate mortality loss term. The value 3000 is a calculation scale, not the observed starting or surviving population. Mean daily feed intake was averaged across the 32 recorded daily values per cage, expressed per rabbit, and multiplied by 33 d and 3000. Mean per-rabbit gain was multiplied by 3000. Mortality was calculated retrospectively among the 99 initially non-implanted rabbits per house, giving 8.08% (8/99) and 9.09% (9/99). Economic calculations used the values displayed to two decimal places in Table 6, including mortality percentages. Combined electricity cost was calculated from the summed fan and pump electricity use. Each monetary component was rounded to two decimals before calculating partial-budget benefits and their difference. The partial-budget benefit was calculated as [10]

*T*_EB_ = *W*_GV_ − (*F*_C_ + *E*_C_ + *M*_L_) (1)

Here, *T*_EB_ is the partial-budget benefit and *W*_GV_ is the value of projected live weight gain. *F*_C_, *E*_C_, and *M*_L_ represent feed cost, combined fan and pump electricity cost, and mortality loss, respectively. All values are in Chinese yuan (CNY). Prices were CNY 0.45/kWh for electricity, CNY 3.00/kg for feed, and CNY 15.00/kg live weight. Mortality loss was calculated as projected deaths multiplied by a common final body weight of 2.16 kg based on the pooled mean of the 152 retained rabbits and the live rabbit price.

The prototype component estimate was CNY 4902.60. Both houses had controllers, and this estimate is not a comparable commercial installed system quotation or the incremental cost of adopting the treatment strategy. Capital, depreciation, maintenance, labor, financing, water, and other farm costs were excluded from the partial-budget analysis. Sensitivity analyses considered prices, mortality inputs, production scale, water recirculation, and electricity use during unmetered days. These scenarios are assumption checks, not estimated confidence limits or a complete farm profit calculation. The difference in partial-budget benefit was calculated as

Δ*T*_EB_ = *T*_EB1_ − *T*_EB2_ (2)

Here, Δ*T*_EB_ is the difference in partial-budget benefit, and *T*_EB1_ and *T*_EB2_ are the treatment and control house estimates, respectively.

**Table 6:** Estimated 33-day resource use in the observed houses and projected production at a standardized scale of 3000 rabbits per house.

Group	Fan Electricity (kWh)	Water Pump Electricity (kWh)	Cooling Pad Recirculation Volume (m^3^)	Projected Feed (kg)	Projected Gain (kg)	Scenario Mortality (%)
Treatment	4265.89	83.77	457.40	11,986.82	3889.05	8.08
Control	3113.19	476.66	2407.11	11,279.02	3593.08	9.09

### 2.5. Data Quality Control and Statistical Analysis

Data were analyzed in Python 3.11.5 and expressed as the mean ± standard deviation (SD). The statistical units and tests are specified below. Statistical analyses used unrounded observations, with final results rounded for presentation.

Environmental records were screened for missing or nonnumeric values, physical validity failures, and sensor-specific values beyond 1.5 interquartile ranges (IQR) from the quartiles. IQR limits were calculated over the complete analysis window. Physical screening ranges were 0.1–50 °C for indoor temperature, 0.1–60 °C for outdoor temperature, 0.1–100% for RH, 0–20 m s^−1^ for air velocity, and 200–5000 ppm for CO_2_. Water vapor pressure was calculated from paired temperature and RH records at each monitoring location using the saturation–vapor pressure relationship of the American Society of Heating, Refrigerating and Air-Conditioning Engineers (ASHRAE).

Out of 770,294 environmental records, 792 (0.103%) were missing or nonnumeric, 1060 (0.138%) failed physical validity screening, and 3689 (0.479%) were IQR outliers. The remaining 764,753 records (99.281%) were retained (Table S3).

Environmental analyses covered 26 July–27 August 2024. For house temperature, relative humidity, and air velocity, valid zone values were averaged at each recorded time before hourly aggregation. House-level CO_2_ was the equal-weight mean of the three zone-specific hourly values using only hours with all three zones available. Environmental daily means required at least 18 valid hours. Body temperature analyses covered 28 July–27 August 2024. Available individual readings were averaged within each zone before calculating daily means. Daily zone means required at least 72 non-missing timepoints. Records from 3, 2, and 2 implanted rabbits contributed to the treatment house cooling pad, central, and fan zones, respectively, compared with 2, 3, and 0 rabbits in the control house (Table S4). Contributions varied over time. Control fan zone records were excluded from the primary series because the early recordings contained abnormal readings and follow-up was incomplete. The between-house summary equally averaged the cooling pad and central zone daily means.

Supplementary Tables S3 and S4 report environmental screening counts and the numbers of original and analyzed body temperature records by house, zone, and anonymized logger. Sensor positions and pretrial comparison checks are described in Section 2.3.

**Figure 5:**
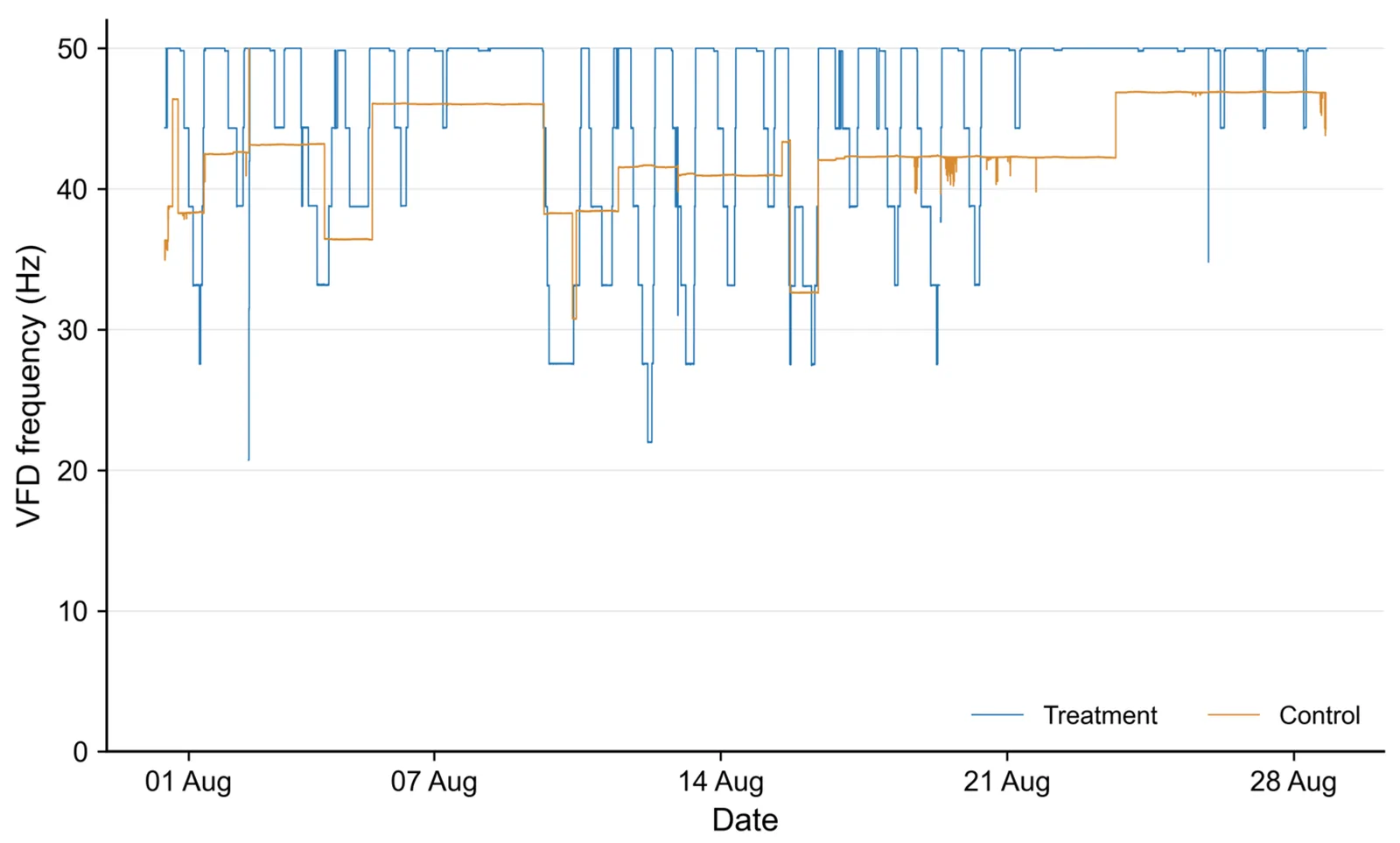
Variable-frequency drive (VFD) operating frequency in the treatment and control houses from 31 July to 28 August 2024.

VFD feedback was recorded at approximately 1 min intervals from 31 July to 28 August 2024. Values outside of 0–100 or corresponding to frequencies below 10 Hz were excluded, with 100 feedback units representing 50 Hz. Paired valid records were plotted without averaging or smoothing, and gaps longer than 2 min were left unconnected (Figure 5).

In the pad, central, and fan zones, respectively, the reconstructed treatment house body temperature series contained 2930/14, 2944/0, and 2930/14 valid/missing timepoints. The corresponding control house counts were 2925/26, 2945/6, and 0/2951. Each available zone series provided 31 days with at least 72 valid timepoints per day. The control fan zone series provided none.

House-level environmental variables and body temperature were compared using same-day treatment minus control differences from 31 to 32 matched days. To account for temporal autocorrelation, the primary analysis used a first-order autoregressive [AR(1)] bootstrap-t procedure using empirical innovations and 49,999 bootstrap replicates. A linear mean was fitted with date centered on the observed dates; the reported difference *D* remained the equally weighted daily mean, not the generalized least squares (GLS)-weighted intercept. A Gaussian quasi-restricted-likelihood criterion estimated covariance parameters without assuming Gaussian innovations in the resampling distribution. Centered, variance-standardized innovations from genuinely consecutive days were resampled; cross-gap innovations were not pooled with daily innovations. Bootstrap errors were generated on the full daily calendar after burn-in, reducing the initial state coefficient below 10^−8^, and then restricted to the original observation dates without imputing observed data. Each replicate re-estimated the covariance parameters and linear mean. Studentization used the model-based standard error of *D* calculated over the original observation dates. Two-sided *p* values and symmetric nominal pointwise 95% intervals used the same absolute bootstrap-t distribution. Table 2 used Holm across four indoor environmental outcomes; Table 5 used one three-test family for cooling pad, central, and equally weighted shared zone body temperature contrasts.

Environmental zone comparisons used 32 common dates across the six house zone groups for each variable. Corresponding zone contrasts used the empirical innovation AR(1) bootstrap-t with Holm across three zones separately for each environmental variable. Uppercase letters reflect these adjusted comparisons at the 0.05 threshold; lowercase letters retain within-house Tukey honestly significant difference (HSD) reference comparisons. These within-house reference comparisons do not account for pairing by date or serial dependence.

Temporal sensitivity checks included daily paired *t*-tests as references and Gaussian innovation AR(1) bootstrap-t with the same centered linear mean (Table S5A). Heteroskedasticity and autocorrelation consistent (HAC) checks used 3 and 7 calendar day lags, each with and without a linear time trend. Paired references used identical retained dates and Holm families; their point intervals used n − 1 degrees of freedom. Screening sensitivity omitted only IQR filtering while retaining physical validity, aggregation, and completeness rules (Table S5B). Each setting used the family of its corresponding primary comparison; settings were not pooled. The primary method assumes a linear mean and stationary AR(1) residuals with independent, identically distributed innovations represented by the empirical pool. With 31–32 days, correlation and tails are uncertain; nominal intervals and *p* values are model-conditional approximations.

Production analyses included 152 rabbits (74 treatment and 78 control) in 76 cages (37 treatment and 39 control). Initial and final weights are reported as individual rabbit mean ± SD, but all inferential analyses used cage-level observations. Body weight observations were the mean of the two rabbits within each cage. Average daily gain (ADG) was cage mean weight gain divided by 33 d. Daily feed intake (DFI) averaged the 32 available daily feed values, and the feed conversion ratio (FCR) was the mean DFI multiplied by 33 d and divided by weight gain. All overall and corresponding zone between-house comparisons used two-sided Welch *t*-tests on cage-level observations. The five overall production outcomes formed one Holm family. For each outcome, its three corresponding zone comparisons formed a separate Holm family. Within-house zone comparisons used Tukey–Kramer. Each Table S2 cage inclusion scenario used these same Welch tests and Holm families. Table 6 retained descriptive accounting without inferential tests. Between-house decisions used unrounded Holm-adjusted *p* values at the 0.05 threshold. Displayed *p* values are rounded to three decimals, with *p* < 0.001 for smaller values.

The comparison families were defined according to the scientific questions addressed by the tables: overall between-house outcomes and spatial differences within each outcome. Holm adjustment controls multiplicity within each stated family; it does not model serial dependence or resolve the absence of independent house replication. Inference remains conditional on the two observed houses. Cages, sensors, and dates do not independently replicate a control strategy.

## 3. Results and Discussion

### 3.1. Indoor Environmental Conditions Under Different Control Strategies

Mean outdoor temperature and relative humidity were 28.88 °C and 82.77%, respectively (Table 2). The treatment house had a mean air temperature of 27.30 ± 1.53 °C versus 26.94 ± 1.37 °C in the control house (Holm-adjusted *p* < 0.001), lower relative humidity (89.68% versus 94.02%, *p* < 0.001), and lower air velocity (0.23 versus 0.35 m s^−1^, *p* = 0.034). The house mean CO_2_ contrast did not meet the adjusted threshold (516.06 versus 533.92 ppm, *p* = 0.612).

The treatment house was 0.36 °C warmer and 4.34 percentage points less humid, indicating a temperature–humidity trade-off. Outdoor conditions may have had limited pad operation when evaporative cooling potential was low [18,19]. Although relative humidity falls as air warms at unchanged moisture content [20], calculated water vapor pressure was also lower in the treatment house (3.26 ± 0.26 versus 3.35 ± 0.28 kPa). These results indicate lower indoor water vapor levels in the treatment house.

Animal populations and production categories differed between houses, with estimated biomass densities of 2.7 and 2.4 kg m^−3^ in the treatment and control houses, respectively. These differences could influence heat, moisture, and CO_2_ production as well as local airflow. They therefore provide alternative explanations for part of the environmental contrast. Because strategy and house identity were confounded, their contributions cannot be separated from the combined fan and pad operating arrangements.

Baseline pad comparability also remains uncertain. In replicated evaporative pad tests using farm well water, mineral accumulation was not accompanied by a significant static pressure increase over one simulated summer [21]. Pad appearance and airflow resistance therefore require separate assessment. Our visual inspection did not establish equivalent pretrial airflow or cooling performance, leaving their contribution to the observed house differences unresolved.

Environmental screening retained 764,753 out of 770,294 records (Table S3). Omitting IQR filtering retained 768,442 records and changed the treatment minus control differences only slightly (Table S5B). Temperature changed from 0.357 to 0.358 °C, RH from −4.338 to −4.341 percentage points, air velocity from −0.121 to −0.114 m s^−1^, and CO_2_ from −17.862 to −17.142 ppm. The air velocity contrast changed most proportionally, but none of the directions reversed. This supports limited sensitivity to IQR rejection in these records; it does not validate sensor calibration. Lag-1 correlations of overall environmental differences ranged from 0.523 to 0.948 (Table S5A). The empirical innovation primary analysis gave overall adjusted *p* values of <0.001, <0.001, 0.034, 0.612 for temperature, RH, air velocity, and CO_2_, respectively. Gaussian innovation and HAC settings changed interval widths and some decisions. Their agreement in direction does not remove short series uncertainty or independently validate the assumed temporal model.

Both houses showed longitudinal temperature and humidity gradients (Table 3), with the lowest temperature near the pads and the highest near the fans. Treatment minus control temperature differences were 0.22, 0.67, and 0.19 °C in pad/central/fan order. Adjusted *p* values were 0.151, <0.001, and 0.005, respectively; the pad contrast did not meet the threshold. RH was numerically lower in all three zones, with adjusted *p* values <0.001, 0.002, and 0.192; the fan contrast did not meet the threshold. Air velocity was lower in the pad and fan zones (*p* = 0.024 and 0.002); the central contrast did not meet the adjusted threshold (*p* = 0.055). Such spatial variation is consistent with the influence of pad and fan placement on temperature and humidity distributions reported in computational fluid dynamics (CFD) simulations of evaporatively cooled greenhouses [22].

Recorded air velocity was lowest near the treatment cooling pad (0.07 m s^−1^) compared with 0.24 m s^−1^ at the corresponding control location. These low values and their difference are small relative to the anemometer accuracy of ±(0.2 + 0.02 v) m s^−1^ and are therefore treated as reference measurements. Pad blockage and other within-house flow resistance can affect fan operating conditions and ventilation rates [23], but blockage was not quantified here. Lower local airflow, if present, could reduce convective cooling and contribute to spatial differences in animal responses.

CO_2_ concentrations varied along both houses, although their house means did not differ significantly. Local air velocity does not measure whole-house ventilation. A CO_2_ balance additionally requires inlet–outlet concentrations and estimates of animal CO_2_ production [24]. Together, the zonal measurements show why house averages should be considered alongside conditions at animal locations [25]. With identical dates and Holm families, the central zone CO_2_ contrast changed from significant in the paired daily reference (*p* = 0.004) to not meeting the threshold in the empirical innovation AR(1) analysis (*p* = 0.946). Gaussian innovation AR(1) and trend-adjusted HAC(7) values were 1.000 and 0.009 (Table S6), showing sensitivity to temporal assumptions. The pad CO_2_ difference remained positive (*p* = 0.012); the fan contrast did not meet the threshold (*p* = 0.946). Near-unit persistence produced very wide intervals for mean contrasts, indicating limited precision under this error model. These are not ranges of daily concentrations, and a nonsignificant result does not establish equivalence.

Available operating frequency records showed more sustained high-frequency fan operation in the treatment house (Figure 5), consistent with its greater fan electricity use. Measured local air velocity was lower, whereas house mean CO_2_ was numerically lower without a significant difference (*p* = 0.612). Operating frequency does not directly quantify ventilation volume. The anemometer accuracy limits resolution of small differences under these low-velocity conditions, so the air velocity measurements provide indicative local information.

### 3.2. Growth Performance and Feed Conversion in the Treatment and Control Houses

Initial body weights were similar between houses (*p* = 0.639, Table 4). Near the treatment cooling pad, final weight, ADG, and DFI averaged 2332.31 ± 155.44 g, 43.03 ± 3.17 g rabbit^−1^ d^−1^ and 130.86 ± 10.02 g rabbit^−1^ d^−1^, respectively. Higher feed intake and growth may reflect differences in local thermal conditions. Chen et al. [26] showed that air distribution influenced convective heat loss in lactating sows. Here, the treatment pad zone was numerically 0.22 °C warmer (Holm-adjusted *p* = 0.151) and 5.40 percentage points less humid (*p* < 0.001) than the control pad zone. Lower humidity may favor evaporative heat loss [12]. Adverse responses to humid conditions have also been reported in dairy cows [27] and ducks [28].

At the house level, final body weight was higher in the treatment house by 103.95 g (pointwise 95% confidence interval: 43.96–163.93 g; Holm-adjusted *p* = 0.004). ADG and DFI were also higher (Holm-adjusted *p* = 0.003 and 0.016, respectively; Table 4). In corresponding zone comparisons, final weight, ADG, and DFI were higher near the treatment cooling pad after per-outcome Holm adjustment (*p* = 0.004, 0.002, and 0.035, respectively).

FCR averaged 3.09 and 3.15 in the treatment and control houses, respectively, and the difference was not significant (Holm-adjusted *p* = 0.581). The numerical reduction was 1.98%, calculated from unrounded means using the control mean as the denominator. Liu et al. [6] likewise reported changes in rabbit feed intake and daily gain without a significant change in FCR, whereas other rabbit populations showed higher FCR under heat stress [29]. The nonsignificant FCR difference does not establish improved feed efficiency.

Whole-cage exclusions left 37 out of 54 treatment cages and 39 out of 54 control cages in the primary production analysis (Table S1; Figure S1). Table S2 distinguishes each treatment minus control contrast (*D*) from its change relative to the primary contrast (Δ*D*). Adding the available diarrhea cage changed the final weight contrast from 103.95 to 95.41 g (Δ*D* = −8.54 g). Adding both available diarrhea- and feed-wastage-affected cages gave 107.05 g (Δ*D* = +3.10 g). The ADG contrasts were 2.990, 2.753, and 3.081 g rabbit^−1^ d^−1^, with changes of −0.236 and +0.091 g rabbit^−1^ d^−1^. The five-outcome Holm-adjusted *p* values for final weight and ADG remained below 0.05 in both reinclusion scenarios. DFI contrasts remained positive, but the adjusted *p* value increased from 0.016 to 0.036 and then 0.062. Thus, the evidence for higher intake was sensitive to the available cage inclusion scenarios. These checks quantify changes in the difference between the two houses; they cannot recover missing endpoints or eliminate selection bias from whole-cage exclusions.

The higher estimated biomass density in the treatment house may have increased heat and moisture production and altered local airflow, potentially influencing growth and feed conversion. Because animal loading and control strategy differed between houses, their contributions to the observed production outcomes cannot be separated.

The mixed-sex sample followed routine commercial management, in which market rabbits were reared together without selection by sex. This commercial setting explains the sampling practice, but recording sex, at least at final weighing, would still have allowed sex balance and sex-specific responses to be assessed. Because sex was not recorded, unequal sex composition could have contributed to the observed variation in growth, intake, and body temperature; neither its presence nor its magnitude can be determined from these records.

Recent work on New Zealand White rabbits reported sex by age differences in growth and body composition despite broadly similar growth patterns between sexes [30]. These findings support recording sex during growth studies, but they do not establish the direction or magnitude of a sex effect in the present Hyla rabbits.

### 3.3. Rabbit Intra-Abdominal Temperature Under Different Environmental Control Strategies

Daily mean intra-abdominal temperature near the cooling pad was 0.29 °C lower in the treatment house (Holm-adjusted *p* = 0.001). The central zone contrast did not meet the adjusted threshold (39.91 versus 39.94 °C, *p* = 0.561). The equally weighted cooling pad and central zone mean was 39.80 ± 0.21 °C versus 39.96 ± 0.26 °C (*p* = 0.013; Table 5). All three contrasts belonged to one Holm family. The 12 contributing logger streams supplied 25,526 readings to the primary zone day means. Table S4 distinguishes these contributions from all 28,857 exported readings across 17 identified loggers. Control fan zone records remained excluded because early readings were abnormal and follow-up was incomplete.

The lower body temperature near the cooling pad coincided with greater growth, consistent with lower thermal strain at this location. In lactating sows, conductive cooling reduced body temperature without significantly increasing litter weight at weaning [31]. Body temperature may also lag behind environmental changes [32]. Daily means do not reveal whether lower peaks or better overnight recovery contributed to the difference. Exclusion of the control fan zone series prevented a between-house physiological comparison at that location. The empirical innovation AR(1) analysis gave differences of −0.289 °C (nominal pointwise 95% interval [−0.397, −0.181]) and −0.162 °C ([−0.253, −0.071]) for cooling pad and shared zone temperatures, respectively. The central zone interval included zero. Table S5A compares the Gaussian innovation and HAC alternatives; differences in interval width reflect temporal model uncertainty. The shared zone mean includes the cooling pad series, so agreement between these contrasts does not independently replicate the physiological response. The spatially limited response cautions against treating the cooling pad finding as a uniform physiological benefit throughout the house. Broader welfare benefits require additional indicators.

### 3.4. Resource Use and Projected Economic Outcomes

Over the 33-day trial, estimated fan electricity use was 1152.70 kWh higher and pump electricity use was 392.89 kWh lower in the treatment house than in the control house (Table 6). Combined electricity use was 759.81 kWh higher, an increase of 21.2%. Fan-only use was approximately 37.0% higher, consistent with the more sustained high-frequency operation in Figure 5. Because fan and pad controls differed together, these electricity differences describe the combined strategies. Advanced control also entails implementation effort, as shown in building control field deployments [33]. Temperature–humidity index-based ventilation simulations likewise linked improved thermal conditions to higher fan loading in livestock housing [34].

Resource values extrapolate measured daily means to 33 d using nominal water and electricity intervals of 28 and 31 d, respectively. Feed and gain apply retained sample means to a standardized scale of 3000 rabbits, whereas resource estimates refer to the observed houses. The mortality penalty is separate and does not reconstruct a starting cohort. Tables S7 and S8 assess the scenario assumptions. *p* values are not reported because there is one resource total per house.

Metered cooling pad recirculation volume was 81.0% lower in the treatment house. Intermittent pumping may have reduced cumulative watering time, while multi-parameter activation may have avoided pad operation when indoor cooling demand or outdoor conditions did not meet the activation criteria. Both mechanisms could have contributed, but their individual effects cannot be separated because activation criteria and watering schedules differed simultaneously between houses. This difference was observed under the combined operating arrangements and cannot be attributed specifically to the controller or watering schedule. Fresh water replenishment and evaporation were not measured, so the result cannot be interpreted as a corresponding reduction in fresh water consumption. Extrapolation to 33 d provides a common temporal basis but assumes the measured mean use also applied to unmetered days. Different weather or equipment operation during those days could change the full-period estimates.

Projected feed consumption and live weight gain were 707.80 and 295.97 kg higher, respectively, in the treatment house scenario. The economic calculation used the Table 6 mortality inputs of 8.08% and 9.09%. At the stated prices, projected gain revenue was CNY 58335.75/53896.20, feed cost was CNY 35960.46/33837.06, combined fan and pump electricity cost was CNY 1957.35/1615.43, and mortality loss was CNY 7853.76/8835.48 (treatment/control). Partial budget benefits were therefore CNY 12564.18 and CNY 9608.23. The additional benefit of CNY 2955.95 represented 30.76% of the control value and 3.04% of benchmark gross sales of CNY 97200 calculated for 3000 rabbits at 2.16 kg before mortality adjustment. The 3.04% describes additional benefit relative to benchmark sales, rather than an increase in sales revenue.

The prototype hardware cost was CNY 4902.60. This component estimate does not establish the incremental installed investment because both houses had controllers and commercial prices include development and operating expenses. The partial budget excludes capital investment, depreciation, maintenance, labor, and water costs. Table S7 evaluates the sensitivity to prices, mortality inputs, and the scaling population. Feed and gain are based on retained cages, with mortality represented by a separate loss term; the calculation is a standardized comparison rather than a complete farm budget.

With equal mortality inputs, the difference in partial-budget benefit decreased from CNY 2955.95 to CNY 1974.23; one additional treatment death and one fewer control death reduced it to CNY 992.42 (Table S7). A ±20% change in live rabbit price produced differences of CNY 1871.69–4040.21 compared with CNY 2531.27–3380.63 for feed price and CNY 2887.57–3024.34 for electricity price. These scenarios show greater sensitivity to mortality and sale price than electricity price over the tested ranges. The CNY 1307.02 and CNY 6253.82 differences at 1500 and 6000 production-equivalents also depend on holding observed house electricity fixed; they are scale assumptions rather than predictions of commercial profitability.

A recent full-cycle meat rabbit model likewise evaluated feed and carcass price scenarios and recommended periodic reassessment of economic weights [35]. The positive partial-budget differences in Table S7 should similarly be interpreted as conditional scenario results. They do not establish an overall farm profit advantage after unmeasured capital, maintenance, labor, and water costs.

When the same unmetered day multiplier was applied to both houses, the relative recirculation and electricity differences remained at −81.0% and +21.2% because both house totals were scaled proportionally (Table S8). Applying the assumptions independently to the houses broadened the estimated recirculation reduction to 74.2–86.0% and the electricity increase to 7.3–36.8%. The directions persisted within these tested scenarios, but the magnitudes depend on unobserved resource use during the missing periods. Recirculated flow remains distinct from freshwater replenishment. The sensitivity analysis supports this resource trade-off under the stated assumptions while leaving freshwater consumption and unmetered day operation unresolved.

### 3.5. Limitations

Each strategy was applied to one fixed house. Differences in animal categories, population, and estimated biomass density are potential confounders, as are unmeasured pretrial pad resistance and the lack of comparable baseline airflow measurements. Visual pad inspection does not establish equivalent baseline performance. Neither statistical adjustment nor the added sensitivity analyses can separate these house differences from the combined control strategy or its components. Independent houses and other seasons and life stages are needed to assess broader applicability.

The short observation period limits estimation of temporal dependence and tail probabilities. The empirical innovation AR(1) model assumes a linear mean and stationary residual dependence; near-boundary correlation estimates and the 30-observation innovation pool make its intervals approximate. For fan zone CO_2_, bootstrap correlation estimates were often high, indicating limited precision in the estimated mean difference. Sensitivity analyses assess dependence on these choices but do not establish exact coverage. All comparisons remain conditional on the observed houses and do not independently replicate a control strategy. Unrecorded sex and whole-cage exclusions introduce further uncertainty, as discussed in Section 3.2. Cage-level inference accounts for paired housing but does not provide house-level replication.

Physiological assessment was limited to intra-abdominal temperature in the available zones. Behavioral and clinical measurements are needed to assess broader welfare benefits.

Whole-cage exclusions restrict production estimates to the retained sample. Table S2 assesses how the available records from diarrhea- and feed-wastage-affected cages influence growth and intake estimates. Scaling these sample means to 3000 rabbits facilitates comparison but does not reconstruct the performance or complete costs of the original population. Deployment costs were not included. Resource extrapolation assumes that measured mean daily use also applied to unmetered days.

## 4. Conclusions

In this 33-day summer field comparison, the multi-parameter control house had lower relative humidity, lower intra-abdominal temperature near the cooling pads, and greater rabbit growth than the conventionally controlled house. Feed conversion did not differ significantly. The treatment house had 81.0% lower estimated cooling pad recirculation volume on the common 33-day scale, while combined fan and pump electricity use was higher. Fresh water savings were not quantified. The partial-budget analysis estimated a benefit CNY 2955.95 higher for the treatment house than for the control house under the stated prices and scaling assumptions; this difference was sensitive to mortality inputs. These preliminary findings apply to the combined fan and pad strategies in the two observed houses. Practical adoption should consider conditions at animal locations and the trade-off between animal performance, resource use, and deployment costs.

## Figures and Tables

**Table 1:** Specifications of the monitoring and weighing devices used in the two rabbit houses.

Sensor Type	Model	Range	Accuracy	Manufacturer
Indoor AT and RH	RS-WS-N01-2D -LCD-5-FD	AT: −40 to 80 °C; RH: 0–100% RH	AT: ±0.2 °C; RH: ±2%	Shandong Renke Measurement and Control Technology Co., Ltd., Jinan, China
Outdoor AT and RH	RS-WS-N01-BYH-FD	AT: −40 to 80 °C; RH: 0–100% RH	AT: ±0.2 °C; RH: ±2%	
Indoor air velocity	RS-CFSFX-N01-2FD	0–60 m s^−1^	±(0.2 + 0.02 v) m s^−1^	
Indoor CO_2_	RS-CO_2_-N01-2	0–5000 ppm	50 ppm + 3% F.S.	
BT	LT-ATDS001	35 to 45 °C	±0.2 °C	Hefei Shenmu Information Technology Co., Ltd., Hefei, China
Weight	LB-07	0–10 kg	±3 g	Longbei Industry and Trade Co., Ltd., Jinhua, China
Single-phase electrical energy meter	DDS6111	Current: 0–100 A	Class 1 for active energy	Zhejiang Zhidian Instruments and Meters Co., Ltd., Wenzhou, China
Three-phase electrical energy meter	DTSU5886	Current: 0–100 A	Class 1 for active energy	Zhejiang Lianyang Electric Co., Ltd., Wenzhou, China
Water meter	LXS-32	0–99,999 m^3^	class 2	Ningbo Yongquan Water Meter Co., Ltd., Ningbo, China

AT, air temperature; RH, relative humidity; BT, body temperature; CO_2_, carbon dioxide; v, measured air velocity (m s^−1^); F.S., full scale of the device’s measurement range; ppm, parts per million.

**Table 2:** Daily environmental conditions in the treatment and control rabbit houses during the summer trial.

Group	Indoor Temperature (°C)	Indoor Relative Humidity (%)	Air Velocity (m s^−1^)	CO_2_ (ppm)	Outdoor Temperature (°C)	Outdoor Relative Humidity (%)
Treatment	27.30 ± 1.53	89.68 ± 1.80	0.23 ± 0.02	516.06 ± 24.37	28.88 ± 1.88	82.77 ± 3.82
Control	26.94 ± 1.37	94.02 ± 1.37	0.35 ± 0.05	533.92 ± 73.33		
*p* value	<0.001	<0.001	0.034	0.612	—	—

Values are mean ± standard deviation (SD) across 32 paired daily summaries. Outdoor measurements share one source and are shown once descriptively. Between-house *p* values use two-sided empirical innovation AR(1) bootstrap-t inference with 49,999 replicates and Holm adjustment across the four indoor outcomes. Nominal pointwise 95% intervals for treatment minus control differences are temperature [0.247, 0.468] °C; RH [−5.083, −3.594] percentage points; air velocity [−0.206, −0.037] m s^−1^; and CO_2_ [−1304.714, 1268.991] ppm. Table S5A,B report temporal and screening sensitivity. These model-conditional approximations do not provide independent house replication. CO_2_, carbon dioxide; ppm, parts per million.

**Table 3:** Daily environmental conditions by longitudinal zone in the treatment and control rabbit houses during the summer trial.

Items	Treatment House			Control House		
	Near the Cooling Pad Area	Central Area	Near the Fan Area	Near the Cooling Pad Area	Central Area	Near the Fan Area
Air temperature (°C)	26.69 ± 1.49 ^bA^	27.54 ± 1.54 ^abA^	27.66 ± 1.58 ^aA^	26.47 ± 1.30 ^bA^	26.87 ± 1.39 ^abB^	27.47 ± 1.43 ^aB^
Relative humidity (%)	91.58 ± 2.57 ^aA^	88.26 ± 1.70 ^bA^	89.20 ± 1.72 ^bA^	96.99 ± 1.35 ^aB^	94.18 ± 1.45 ^bB^	91.09 ± 1.22 ^cA^
Air velocity (m s^−1^)	0.07 ± 0.02 ^cA^	0.33 ± 0.04 ^aA^	0.30 ± 0.03 ^bA^	0.24 ± 0.09 ^cB^	0.37 ± 0.05 ^bA^	0.46 ± 0.04 ^aB^
CO_2_ (ppm)	504.85 ± 25.40 ^bA^	499.38 ± 23.98 ^bA^	545.00 ± 40.21 ^aA^	456.04 ± 15.64 ^bB^	551.72 ± 84.14 ^aA^	593.99 ± 130.85 ^aA^

Values are mean ± standard deviation (SD) across 32 common dates. Lowercase letters show reference within-house Tukey HSD comparisons and do not incorporate serial dependence. Uppercase letters use corresponding zone empirical innovation AR(1) bootstrap-t comparisons with Holm adjustment across the three zones separately for each variable. Different letters indicate adjusted *p* < 0.05. Holm-adjusted between-house *p* values in cooling pad/central/fan order: temperature, 0.151/<0.001/0.005; RH, <0.001/0.002/0.192; air velocity, 0.024/0.055/0.002; CO_2_, 0.012/0.946/0.946. Between-house inference is model-conditional on the observed houses. Corresponding zone nominal pointwise 95% intervals for treatment minus control differences in cooling pad/central/fan order: temperature, [−0.132, 0.565]/[0.539, 0.795]/[0.086, 0.292] °C; RH, [−6.832, −3.973]/[−8.174, −3.655]/[−5.787, 1.994] percentage points; air velocity, [−0.281, −0.056]/[−0.087, 0.001]/[−0.216, −0.096] m s^−1^; CO_2_, [24.833, 72.787]/[−792.157, 687.466]/[−37808.767, 37710.770] ppm. Alternative temporal settings are in Table S6.

**Table 4:** Growth performance of rabbits by house and longitudinal zone during the trial.

Items	Treatment House			Control House			*p* Value
	Near the Cooling Pad Area	Central Area	Near the Fan Area	Near the Cooling Pad Area	Central Area	Near the Fan Area	House
Initial weight (g)	912.31 ± 70.44 ^aA^	922.69 ± 58.96 ^aA^	902.73 ± 62.04 ^aA^	906.82 ± 59.71 ^aA^	909.64 ± 54.80 ^aA^	906.79 ± 59.38 ^aA^	0.639
Final body weight (g)	2332.31 ± 155.44 ^aA^	2142.31 ± 155.78 ^bA^	2143.64 ± 134.96 ^bA^	2149.09 ± 150.77 ^aB^	2063.21 ± 175.46 ^aA^	2113.57 ± 130.13 ^aA^	0.004
Average daily gain (g d^−1^)	43.03 ± 3.17 ^aA^	36.96 ± 2.55 ^bA^	37.60 ± 2.34 ^bA^	37.64 ± 3.46 ^aB^	34.96 ± 3.74 ^aA^	36.57 ± 2.73 ^aA^	0.003
Daily feed intake (g d^−1^)	130.86 ± 10.02 ^aA^	116.47 ± 6.12 ^bA^	114.96 ± 8.53 ^bA^	119.10 ± 10.71 ^aB^	110.73 ± 10.62 ^aA^	113.07 ± 9.97 ^aA^	0.016
Feed conversion ratio	3.05 ± 0.20 ^aA^	3.16 ± 0.22 ^aA^	3.06 ± 0.19 ^aA^	3.17 ± 0.27 ^aA^	3.19 ± 0.39 ^aA^	3.10 ± 0.23 ^aA^	0.581

Values are mean ± standard deviation (SD) of individual rabbits for initial/final weight and of cage-level observations for ADG, DFI, and FCR. Treatment pad/central/fan samples were 26/26/22 rabbits in 13/13/11 cages; control samples were 22/28/28 rabbits in 11/14/14 cages. All between-house tests were two-sided Welch *t*-tests on cage-level observations. Lowercase letters denote within-house Tukey–Kramer comparisons. Uppercase letters use Holm adjustment across the three corresponding zone comparisons separately within each outcome. The house *p* column is Holm-adjusted across the five overall outcomes. Holm-adjusted corresponding zone *p* values in cooling pad/central/fan order: initial weight, 1.000/1.000/1.000; final weight, 0.004/0.194/0.452; ADG, 0.002/0.232/0.319; DFI, 0.035/0.194/0.614; FCR, 0.621/1.000/1.000. Different letters indicate *p* < 0.05 under the corresponding procedure. Comparisons are exploratory and conditional on these houses. ADG, average daily gain; DFI, daily feed intake; FCR, feed conversion ratio.

**Table 5:** Daily mean intra-abdominal temperature of rabbits by house and longitudinal zone.

Zone	Treatment House (°C)	Control House (°C)	*p* Value
Near the cooling pad area	39.70 ± 0.23	39.99 ± 0.31	0.001
Central area	39.91 ± 0.21	39.94 ± 0.24	0.561
Near the fan area	40.07 ± 0.36	N/A	N/A
Mean of cooling pad and central zones	39.80 ± 0.21	39.96 ± 0.26	0.013

Values are mean ± SD across 31 common dates (28 July–27 August); daily means require ≥72 non-missing timepoints. The shared zone mean equally weights pad and central zones. Two-sided empirical innovation AR(1) bootstrap-t comparisons use one three-test Holm family. Nominal pointwise 95% intervals (treatment minus control) are cooling pad [−0.397, −0.181]; central [−0.209, 0.140]; shared zone [−0.253, −0.071]; all °C. Alternative settings retain that family separately (Table S5A). N/A, not analyzed. Inference is conditional on the temporal model and these houses.

## Data Availability

The underlying farm records are not publicly available because of the confidentiality agreement with the collaborating farm. Summary results and analytical procedures are reported in the manuscript and Supplementary Materials.

## Supplementary Materials

The following supporting information can be downloaded at https://www.mdpi.com/article/10.3390/ani16213450/s1, Figure S1: Primary exclusion categories and recorded events by house and longitudinal zone; Table S1: Animal and cage flow by house and zone; Table S2: Between-house production contrasts and their changes under alternative cage inclusion; Table S3: Environmental quality control by house, location and variable; Table S4: Implanted-temperature logger records and contribution to the primary analysis; Table S5A: Temporal sensitivity of same-date environmental and body-temperature comparisons; Table S5B: House-level environmental comparisons with and without IQR filtering; Table S6: Temporal sensitivity of corresponding-zone environmental comparisons; Table S7: Sensitivity of the scaled valid-sample partial-budget analysis; Table S8: Sensitivity to assumed use during unmetered days.

## Abbreviations

The following abbreviations are used in this manuscript:

AC	Alternating current
ADG	Average daily gain
AT	Air temperature
BT	Body temperature
CFD	Computational fluid dynamics
CNY	Chinese yuan
CO_2_	Carbon dioxide
DC	Direct current
DFI	Daily feed intake
FCR	Feed conversion ratio
F.S.	Full scale
HMI	Human–machine interface
IQR	Interquartile range
MQTT	Message Queuing Telemetry Transport
PLC	Programmable logic controller
RH	Relative humidity
RS-485	Recommended Standard 485
RTU	Remote terminal unit
SD	Standard deviation
TCP/IP	Transmission Control Protocol/Internet Protocol
VFD	Variable-frequency drive
4G	Fourth-generation mobile communication
ASHRAE	American Society of Heating, Refrigerating and Air-Conditioning Engineers
GB	Gigabyte
N/A	Not analyzed
ppm	Parts per million
