# Supplementary material for: A Two-House Field Comparison of Multi-Parameter and Conventional Environmental Control for Rabbits Under Hot–Humid Summer Conditions
Source: Animals (Basel). 2026 Nov 1;16(21):3450. doi: 10.3390/ani16213450 (PMC13761676; doi:10.3390/ani16213450)
Supplement: Supplementary file 1 [file animals-16-03450-s001.zip › animals-4626161-supplementary.pdf]

Supplementary materials

Table S1. Animal and cage flow by house and zone

House / zone	Initial cages / rabbits	Implant cages	Nonimplant death cages	Diarrhoea cages	Feed-wastage cages	Retained cages / rabbits
Treatment / Cooling pad	18 / 36	2	2	0	1	13 / 26
Treatment / Central	18 / 36	2	2	0	1	13 / 26
Treatment / Fan	18 / 36	2	4	1	0	11 / 22
Control / Cooling pad	18 / 36	2	4	0	1	11 / 22
Control / Central	18 / 36	2	2	0	0	14 / 28
Control / Fan	18 / 36	2	2	0	0	14 / 28

Note. Exclusions are mutually exclusive, in the order implantation, nonimplanted death, diarrhoea and feed wastage. Counts refer to whole cages, including cage partners.

Figure S1. Primary exclusion categories and recorded events by house and longitudinal zone

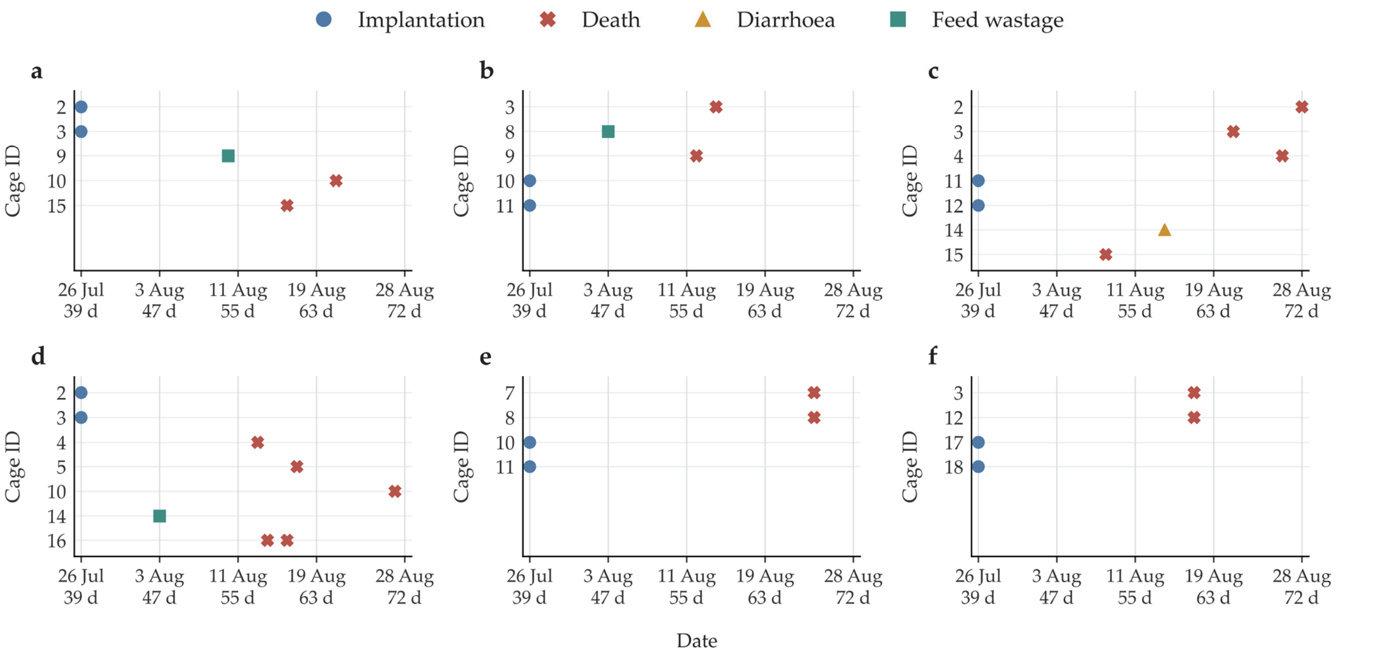

(a) Treatment cooling-pad zone; (b) treatment central zone; (c) treatment fan zone; (d) control cooling-pad zone; (e) control central zone; (f) control fan zone. Each row represents one excluded cage. The two markers for control cooling-pad cage 16 represent two deaths in one excluded cage.

Table S2. Between-house production contrasts and their changes under alternative cage inclusion.

Analysis set and quantity	Cages T / C	Final weight (g)	ADG (g rabbit^{-1} d^{-1})	DFI (g rabbit^{-1} d^{-1})	FCR
Retained sample: *D* (Holm *p*)	37 / 39	103.95 (0.004)	2.990 (0.003)	7.149 (0.016)	-0.063 (0.581)
Add diarrhoea cage: *D* (Holm *p*)	38 / 39	95.41 (0.011)	2.753 (0.010)	6.518 (0.036)	-0.060 (0.620)
Add diarrhoea and feed-wastage cages: *D* (Holm *p*)	38 / 40	107.05 (0.006)	3.081 (0.006)	6.021 (0.062)	-0.125 (0.313)
Change after adding diarrhoea: Δ*D*	38 / 39	-8.54	-0.236	-0.631	+0.003
Change after adding both: Δ*D*	38 / 40	+3.10	+0.091	-1.129	-0.063

Note. *D* is the treatment-house mean minus the control-house mean under the stated cage-inclusion scenario. Values in parentheses are Holm-adjusted *p* values from two-sided Welch t-tests on cage-level observations. The five overall outcomes, including initial weight, form one Holm family within each scenario. Corresponding-zone tests use three-zone Holm families separately for each outcome, matching Table 4. Δ*D* = *D*scenario − *D*primary quantifies how changing cage inclusion changes the difference between the two houses; it is not a within-house change. The primary Δ*D* is zero. All contrasts and changes were calculated before rounding. ADG, average daily gain; DFI, daily feed intake; FCR, feed conversion ratio; T/C, treatment/control.

Table S3. Environmental quality control by house, location and variable

House	Location	Variable	Raw N	Missing / nonnumeric	Physical range	IQR	Retained
Treatment	Pad	AT (°C)	46873	99	9	0	46765
Treatment	Central	AT (°C)	46873	99	9	3	46762
Treatment	Fan	AT (°C)	46873	99	9	0	46765
Treatment	Pad	RH (%)	46873	99	9	172	46593
Treatment	Central	RH (%)	46873	99	9	174	46591
Treatment	Fan	RH (%)	46873	99	9	387	46378
Treatment	Pad	AV (m s^{-1})	46913	0	121	1108	45684
Treatment	Central	AV (m s^{-1})	46913	0	120	671	46122
Treatment	Fan	AV (m s^{-1})	46913	0	123	329	46461
Treatment	Pad	CO_2 (ppm)	46913	0	124	129	46660
Treatment	Central	CO_2 (ppm)	46913	0	124	21	46768
Treatment	Fan	CO_2 (ppm)	46913	0	123	234	46556
Control	Pad	AT (°C)	9486	0	24	0	9462
Control	Central	AT (°C)	9486	0	21	0	9465
Control	Fan	AT (°C)	9486	0	22	0	9464
Control	Pad	RH (%)	9486	0	24	257	9205
Control	Central	RH (%)	9486	0	21	49	9416
Control	Fan	RH (%)	9486	0	22	29	9435
Control	Pad	AV (m s^{-1})	9486	0	19	26	9441
Control	Central	AV (m s^{-1})	9486	0	19	47	9420
Control	Fan	AV (m s^{-1})	9486	0	19	43	9424
Control	Pad	CO_2 (ppm)	9486	0	22	4	9460
Control	Central	CO_2 (ppm)	9486	0	20	6	9460
Control	Fan	CO_2 (ppm)	9486	0	20	0	9466
Treatment	Outdoor	AT (°C)	46873	99	9	0	46765
Treatment	Outdoor	Outdoor relative humidity (%)	46873	99	9	0	46765

Note. AT, air temperature; RH, relative humidity; AV, air velocity; IQR, interquartile-range screening; CO_2, carbon dioxide; ppm, parts per million. Raw N denotes sensor–variable observations before screening, rather than independent experimental replicates. The usual indoor logging interval was 1 min in the treatment house and 5 min in the control house, accounting for the approximately fivefold difference in corresponding raw counts. The main environmental comparisons use 32 paired daily summaries.

Table S4. Implanted-temperature logger records and contribution to the primary analysis

ID	House	Location	Raw N	Final N	Range (°C)	Analysis status
1	Treatment	Pad	2920	2920	37.99–40.78	Included
2	Treatment	Pad	257	257	39.28–40.07	Included
3	Treatment	Pad	2240	2240	36.67–40.32	Included
4	Treatment	Central	1554	0	25.00–39.87	Excluded
5	Treatment	Central	2943	2943	38.54–40.99	Included
6	Treatment	Central	2941	2941	38.16–40.96	Included
7	Treatment	Fan	67	0	39.67–40.21	Excluded
8	Treatment	Fan	2930	2930	37.93–41.68	Included
9	Treatment	Fan	542	542	38.66–40.27	Included
10	Control	Pad	772	772	38.61–41.33	Included
11	Control	Pad	2921	2921	38.72–41.15	Included
12	Control	Central	2941	2941	38.02–40.67	Included
13	Control	Central	2930	2930	38.47–41.58	Included
14	Control	Central	1189	1189	39.22–40.92	Included
15	Control	Fan	467	0	29.22–34.40	Excluded
16	Control	Fan	832	0	37.85–40.49	Excluded
17	Control	Fan	411	0	39.49–40.58	Excluded

Note. Raw N is the number of exported readings; Final N is the number contributing to primary zone-day means with at least 72 valid timepoints. Ranges describe raw readings. One of the 18 rabbits selected for implantation had no identifiable export.

Table S5A. Temporal sensitivity of same-date environmental and body-temperature comparisons.

Outcome	Paired days	Mean Δ	Lag-1 r	Primary 95% CI	Primary Holm *p*	Gaussian Holm *p*	HAC(3) Holm *p*	HAC(7) Holm *p*
Air temperature (°C)	32	0.357	0.523	[0.247, 0.468]	<0.001	<0.001	<0.001	<0.001
RH (%)	32	-4.338	0.777	[−5.083, −3.594]	<0.001	<0.001	<0.001	<0.001
Air velocity (m s^{-1})	32	-0.121	0.798	[−0.206, −0.037]	0.034	0.033	<0.001	<0.001
CO_2 (ppm)	32	-17.862	0.948	[−1304.714, 1268.991]	0.612	0.635	0.190	0.265
Body temperature / Pad	31	-0.289	0.449	[−0.397, −0.181]	0.001	<0.001	<0.001	<0.001
Body temperature / Central	31	-0.034	0.562	[−0.209, 0.140]	0.561	0.561	0.389	0.390
Body temperature / shared-zone mean	31	-0.162	0.475	[−0.253, −0.071]	0.013	0.006	<0.001	<0.001

Note. Δ is the equally weighted treatment-minus-control daily difference. AR(1), first-order autoregressive; HAC, heteroskedasticity and autocorrelation consistent; CI, confidence interval. Primary denotes empirical-innovation AR(1) bootstrap-t with 49,999 replicates and a centred linear mean; Gaussian uses Gaussian innovations with the same covariance fitting and estimand. HAC(3) and HAC(7) retain the centred linear mean and use 3- and 7-calendar-day lags. Every setting applies Holm separately across the four overall indoor environmental outcomes or the three body-temperature contrasts. Different settings are not pooled. The displayed interval is nominal pointwise 95% and model-conditional.

Table S5B. House-level environmental comparisons with and without IQR filtering.

Outcome	Paired days	Original difference	No-IQR difference	Change	No-IQR primary 95% CI	Holm p
Air temperature (°C)	32	0.357	0.358	0.000	[0.247, 0.468]	<0.001
RH (%)	32	-4.338	-4.341	-0.002	[−5.060, −3.621]	<0.001
Air velocity (m s^{-1})	32	-0.121	-0.114	0.008	[−0.172, −0.055]	0.008
CO_2 (ppm)	32	-17.862	-17.142	0.720	[−1450.232, 1415.947]	0.618

Note. Only interquartile-range (IQR) filtering was removed; physical-validity screening, aggregation and daily completeness rules were retained. Differences are treatment minus control. Intervals and p values use the same empirical-innovation AR(1) bootstrap-t as the primary analysis, with Holm across the four overall indoor outcomes. Every screening and temporal setting uses this family separately. Changes are calculated before rounding. Intervals are nominal pointwise 95%, conditional on the temporal model and observed houses.

Table S6. Temporal sensitivity of corresponding-zone environmental comparisons.

Outcome and location	Paired days	Mean Δ	Primary 95% CI	Primary Holm p	Gaussian Holm p	HAC(7) Holm p
Air temperature (°C) / Pad	32	0.217	[−0.132, 0.565]	0.151	0.149	0.021
Air temperature (°C) / Central	32	0.667	[0.539, 0.795]	<0.001	<0.001	<0.001
Air temperature (°C) / Fan	32	0.189	[0.086, 0.292]	0.005	0.005	<0.001
RH (%) / Pad	32	-5.403	[−6.832, −3.973]	<0.001	<0.001	<0.001
RH (%) / Central	32	-5.915	[−8.174, −3.655]	0.002	0.003	<0.001
RH (%) / Fan	32	-1.897	[−5.787, 1.994]	0.192	0.193	<0.001
Air velocity (m s^{-1}) / Pad	32	-0.168	[−0.281, −0.056]	0.024	0.026	<0.001
Air velocity (m s^{-1}) / Central	32	-0.043	[−0.087, 0.001]	0.055	0.056	<0.001
Air velocity (m s^{-1}) / Fan	32	-0.156	[−0.216, −0.096]	0.002	0.003	<0.001
CO_2 (ppm) / Pad	32	48.810	[24.833, 72.787]	0.012	0.013	<0.001
CO_2 (ppm) / Central	32	-52.346	[−792.157, 687.466]	0.946	1.000	0.009
CO_2 (ppm) / Fan	32	-48.998	[−37808.767, 37710.770]	0.946	1.000	0.160

Note. Δ is the equally weighted treatment-minus-control daily difference. Primary is empirical-innovation AR(1) bootstrap-t (49,999 replicates); Gaussian and HAC(7) retain the centred linear mean. The missing date is retained without observational imputation. Each setting applies Holm across three corresponding-zone contrasts separately for each environmental variable; settings are not pooled. Intervals are nominal pointwise 95% and model-conditional.

Table S7. Sensitivity of the scaled valid-sample partial-budget analysis

Assumption changed from reference	Treatment − control partial-budget benefit (CNY per 33 d)
Reference, 3000 valid-sample production-equivalents	2955.95
Live-rabbit price 12 or 18 CNY kg^{-1} (−20% / +20%)	1871.69 / 4040.21
Feed price 2.4 or 3.6 CNY kg^{-1} (−20% / +20%)	3380.63 / 2531.27
Electricity price 0.36 or 0.54 CNY kWh^{-1} (−20% / +20%)	3024.34 / 2887.57
Equal mortality inputs, both 8.08%	1974.23
One more treatment and one fewer control death (9/99 vs 8/99)	992.42
One fewer treatment and one more control death (7/99 vs 10/99)	4919.68
1500 or 6000 valid-sample production-equivalents, house electricity fixed	1307.02 / 6253.82

Note. CNY, Chinese yuan. Reference: 3000 valid-sample production-equivalents; live weight 15 CNY kg^{-1}, feed 3 CNY kg^{-1} and electricity 0.45 CNY kWh^{-1}. Mortality inputs are 8.08%/9.09% (treatment/control). Each row changes the stated assumption(s). Observed-house electricity is fixed in scale scenarios. Values are rounded to two decimals.

Table S8. Sensitivity to assumed use during unmetered days

Unmetered-day assumption	Recirculation T / C (m^3)	Recirculation change (%)	Electricity T / C (kWh)	Electricity change (%)
Zero, both houses	388.10 / 2042.40	−81.0	4086.04 / 3372.28	+21.2
Measured daily mean, both houses (reference)	457.40 / 2407.11	−81.0	4349.66 / 3589.85	+21.2
Twice measured daily mean, both houses	526.71 / 2771.83	−81.0	4613.27 / 3807.41	+21.2
Treatment twice / control zero	526.71 / 2042.40	−74.2	4613.27 / 3372.28	+36.8
Treatment zero / control twice	388.10 / 2771.83	−86.0	4086.04 / 3807.41	+7.3

Note. Unmetered-day use is expressed relative to each house's measured daily mean. Nominal measured intervals are 28 d for recirculation and 31 d for electricity; estimates cover 33 d. T/C, treatment/control. The water quantity is recirculated flow, not freshwater consumption.
